# Disentangling and Quantifying Mechanisms of Increased Fall Risk Among Older Adults With Depression

**DOI:** 10.1093/geroni/igaa057.852

**Published:** 2020-12-16

**Authors:** Matthew Lohman, Nicholas Resciniti, Anwar Merchant

**Affiliations:** University of South Carolina, Columbia, South Carolina, United States

## Abstract

Background: Older adults with depression are up to four times more likely to fall, yet the mechanisms by which depression increases fall risk are unclear. This study sought to quantify and compare the relative strength of selected mechanisms mediating the association between depression and falls. Methods: We used longitudinal linked data (2006 – 2010) from the Health and Retirement Study and Prescription Drug Study. The analytic sample included non-institutionalized adults age > 65 with data on physical functioning and medication use (n=3,565). Falls and injurious falls over the past two years were self-reported outcomes. Depression was measured using the Composite International Diagnostic Interview (CIDI). We used causal mediation analysis to estimate the total and direct associations between depression and falls and compared strength of three potential mediating mechanisms – frailty, cognition, and antidepressant (AD) use. Results: Of 190 participants reporting depression in 2006, 85 (44.7%) fell and 30 (15.8%) were injured from a fall between 2008 and 2010. Depressed individuals were 92% more likely to fall compared to non-depressed (OR=1.92, p<.01). We found significant indirect effects of AD use (indirect OR=1.17, p<.01) and frailty (OR=1.12, p=.013) representing 19% and 13% of the total effect of depression on falls, respectively. Cognition was not a significant mediator. Results were similar for falls leading to injury. Discussion: Results suggest that AD use and frailty explain a significant portion of the elevated risk for falls among depressed individuals. Identification of these and other mechanisms may inform clinical treatment decisions for older adults with depression.

